# Methotrexate-related lymphoproliferative disorder of the stomach in a patient with rheumatoid arthritis: a case of disease regression after methotrexate cessation

**DOI:** 10.1007/s12328-015-0624-5

**Published:** 2016-01-05

**Authors:** Kazuki Ikeda, Takefumi Nakamura, Takahiro Kinoshita, Mikio Fujiwara, Suguru Uose, Hitoshi Someda, Takashi Miyoshi, Katsuhiro Io, Ken-ichi Nagai

**Affiliations:** Department of Gastroenterology and Hepatology, Kansai Denryoku Hospital, 2-1-7 Fukushima, Fukushima-ku, Osaka, 553-0003 Japan; Department of Hematology, Kansai Denryoku Hospital, 2-1-7 Fukushima, Fukushima-ku, Osaka, 553-0003 Japan; Asahibashi Ichoka Naika Clinic, 4-2-26 Nishikujyo, Konohana-ku, Osaka, 554-0012 Japan; Kansai Electric Power Medical Research Institute, 2-1-7 Fukushima, Fukushima-ku, Osaka, 553-0003 Japan

**Keywords:** Rheumatoid arthritis (RA), Methotrexate (MTX), Lymphoproliferative disorder (LPD), Stomach

## Abstract

We report the case of a 78-year-old woman with methotrexate-related gastric lymphoproliferative disorder (LPD). The patient had a history of rheumatoid arthritis (RA) and had been treated with methotrexate (MTX). Endoscopic examination revealed round elevated lesions in the stomach, and a biopsy specimen showed atypical lymphoid cell proliferation. Immunohistological study found these atypical cells to be positive for L-26 but not for CD3 or EBER. Therefore, we made a diagnosis of MTX-related LPD showing features of diffuse large B-cell lymphoma. Combined positron emission tomography-computed tomography (PET-CT) using 18F-fluorodeoxyglucose (FDG) showed increased avidity in the stomach in addition to slightly increased FDG-avidity in the mediastinum and left chest wall. We decided not to start chemotherapy but to discontinue administration of MTX, with follow-up using endoscopy and PET-CT. The endoscopic examinations after cessation of MTX demonstrated gradual regression of the elevated lesions. PET-CT 6 months after cessation showed no increased FDG avidity in the stomach. While disease regression was observed in the stomach, the other FDG-avid spots remained unchanged on PET-CT. Therefore, we performed chemotherapy as additional therapy. On PET-CT after chemotherapy, the FDG-avid spots remained unchanged for more than 1 year, and we eventually concluded that they were RA-related inflammatory lesions. In patients with MTX-related LPD, cessation of MTX may be a therapeutic option, but careful follow-up and chemotherapy in accordance with the clinical course are essential.

## Introduction

Treatment of rheumatoid arthritis (RA) has undergone a revolution in recent years. Methotrexate (MTX), with or without biologics, is the current first-line therapy for RA, and MTX is widely administered as an anchor drug [1]. In patients with RA, lymphoproliferative disorders (LPD) develop at a frequency 2.0–5.5 times higher than in the general population, and MTX has been described as a major cause of LPD [2]. The World Health Organization (WHO) classification of lymphoid neoplasm describes MTX-related LPD as “iatrogenic immunodeficiency-associated LPD”, which is similar to immunodeficiency-associated LPD, including post-transplant LPD and human immunodeficiency (HIV)-associated LPD [3]. Approximately 40–50 % of MTX-related LPD occurs in extra-nodal sites, with the gastrointestinal tract, skin, liver, lung, and kidney reported to be susceptible [4, 5]. Though MTX-related LPD is an uncommon medical condition, its clinical importance in the gastrointestinal area will increase in accordance with an increasing number of cases of RA treated with MTX.

## Case report

**Fig. 1:**
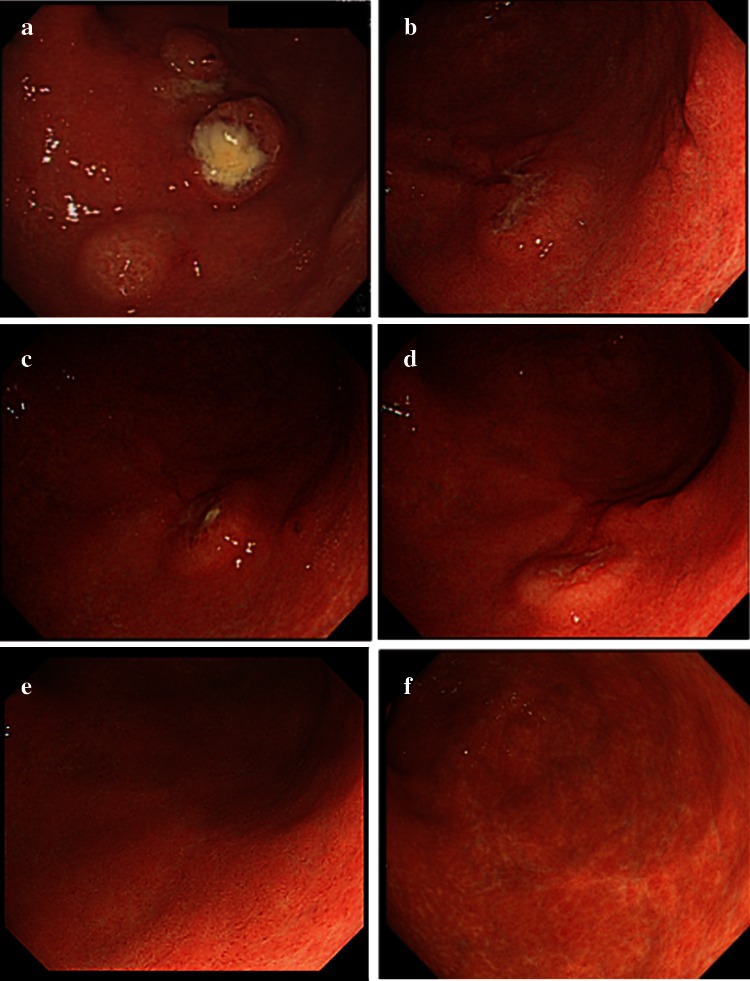
Endoscopic appearance of multiple elevated lesions in the lower body of the stomach **a** before cessation of MTX; **b** 2 weeks, **c** 1 month, **d** 2 months, and **e** 5 months after cessation of MTX; and **f** 1 year after chemotherapy. Regression of the elevated lesion is demonstrated

**Fig. 2:**
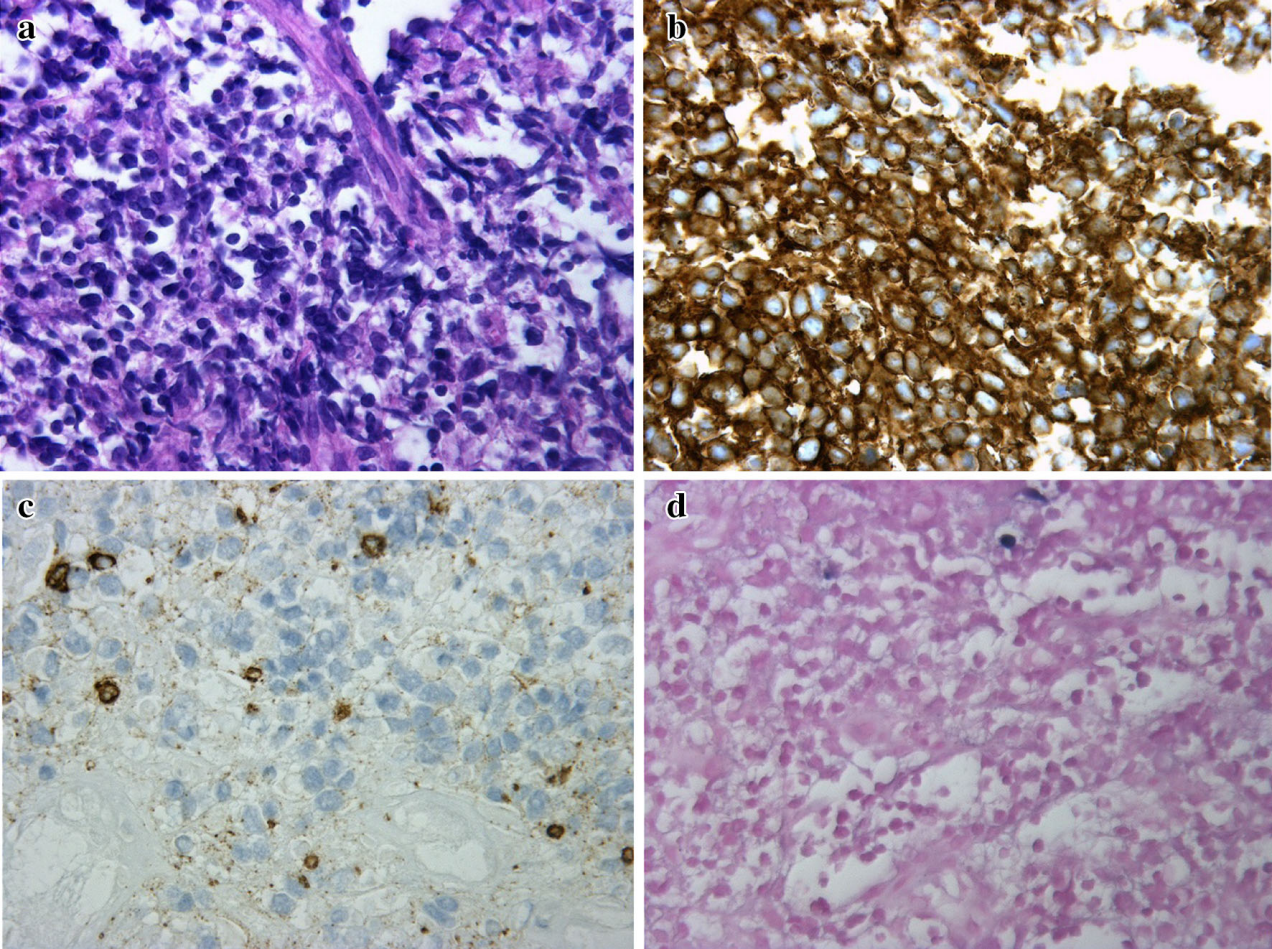
**a** Histopathological features of the ulcerative lesion in the stomach, with proliferation of large-sized lymphoid cells. H&E, ×400. **b** Immunohistological findings of the ulcerative lesion, showing that L-26 was expressed in the large lymphoid cells (×400), and **c** CD3 was not expressed ×400. **d** Few EBER-positive cells were detected by in situ hybridization ×400

**Fig. 3:**
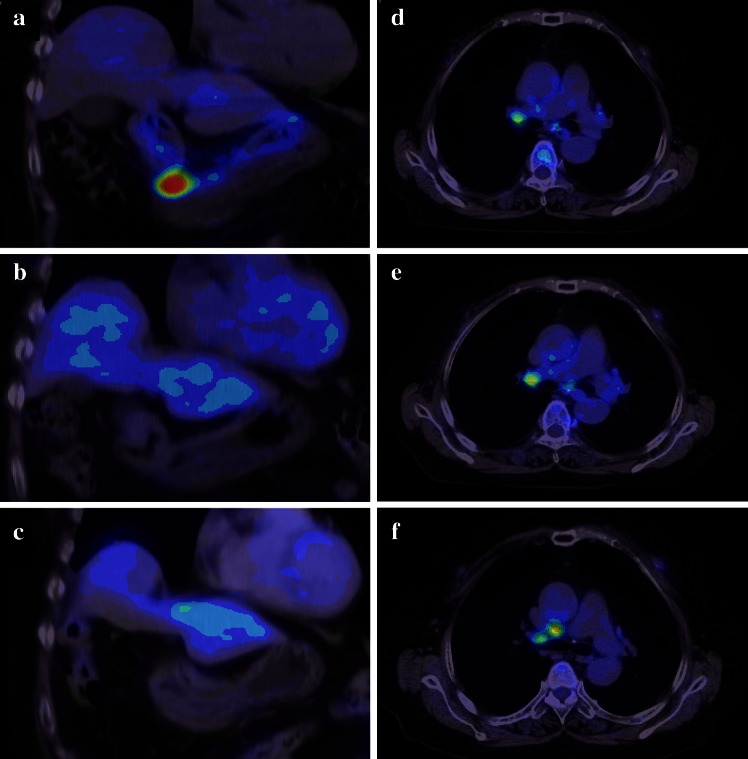
**a**–**c** Coronal PET-CT scan image of the stomach. **d**–**f** Axial PET-CT scan image of the mediastinum and chest wall. **a** Before cessation of MTX, increased FDG avidity was shown in the stomach wall. **b** Six months after cessation of MTX, no FDG avidity was seen in the stomach wall. **c** One year after chemotherapy, no FDG avidity was shown in the stomach wall. **d** Before cessation of MTX, slightly increased FDG avidity was visible in the mediastinum and left chest wall. **e** Six months after cessation of MTX, the slightly increased FDG avidity in the mediastinum and left chest wall remained unchanged.** f** One year after chemotherapy. Slightly increased FDG avidity in the mediastinum and left chest wall remained unchanged.

A 78-year-old woman was referred to our hospital with suspected diffuse large B-cell lymphoma (DLBCL) of the stomach. She had an 18-year history of RA and had been treated with MTX (6 mg/week) for more than 2 years. She complained of slight abdominal fullness after meals and had no other symptoms such as morning stiffness, fever, weight loss, or night sweats. Physical examination revealed neither lymphadenopathy nor hepatosplenomegaly. Laboratory tests revealed normal blood cell count, and slightly elevated soluble interleukin-2 receptor (599 U/ml; range 145–519). Lactate dehydrogenase was within normal range (187 IU/L; 119–229). The test for anti-HCV antibody was positive, whereas HCV-RNA was negative by reverse transcription-polymerase chain reaction (RT-PCR). Urea breath test indicated positivity for *Helicobacter pylori* infection (Otsuka, Japan). An upper endoscopy that had been performed at another clinic revealed multiple round elevated lesions in the middle body of the stomach, similar to submucosal tumors (Fig. 1a). One of them was ulcerated, and endoscopic biopsy was performed at the ulcerated site. Biopsy specimens of the elevated lesion revealed atypical lymphoid cell proliferation, and immunohistological tests showed that the large lymphoid cells were positive for L-26, bcl-6, and bcl-2, but negative for CD10 and CD3. In situ hybridization for EBV (EBER) demonstrated no positivity in the majority of atypical lymphoid cells (Fig. 2a–d). Combined positron-emission tomography and computed tomography (PET-CT) using 18F-fluorodeoxyglucose (FDG) showed increased FDG avidity in the stomach (Fig. 3a) in addition to slightly increased avidity in the mediastinum and left chest wall (Fig. 3d). As the patient had been treated with MTX for RA, we made a diagnosis of MTX-related lymphoproliferative disorder (LPD) showing features of DLBCL. We decided to discontinue MTX, and planned follow-up using endoscopy and PET-CT. Prednisolone and salazosulfapyridine were administered continuously as therapy for RA after cessation of MTX.

Follow-up endoscopy was performed 2 weeks and 1, 2, and 5 months after cessation of MTX, which clearly demonstrated gradual regression of the elevated lesions (Fig. 1b–e), and atypical lymphoid cell proliferation was absent in each biopsy specimen. Moreover, the follow-up PET-CT 6 months after cessation showed no increased FDG avidity in the stomach (Fig. 3b). While disease regression was observed in the stomach, the FDG-avid spots in the mediastinum and left chest wall remained unchanged (Fig. 3e). Subsequently, we performed ultrasonography-guided fine needle aspiration biopsy of the left chest wall nodule, which showed no abnormal lymphoid cell proliferation. Nevertheless, we could not completely rule out the possibility of residual lesions after cessation of MTX, and performed chemotherapy, six cycles of R-THP-COP, consisting of rituximab (375 mg/m^2^, day 1), pirarubicin (30 mg/m^2^, day 1), vincristine (1.4 mg/body, day 1), cyclophosphamide (500 mg/m^2^, day 1), and prednisolone (30 mg/m^2^, day 1–5) every 21 days. THP-COP is a widely used chemotherapy regimen for elderly patients with malignant lymphoma, especially in Japan, in which pirarubicin is chosen in place of doxorubicin used in the standard CHOP regimen.

Endoscopic examination 1 year after chemotherapy showed completely healed white scars in the lower body of the stomach (Fig. 1f), and no disease recurrence was found. After cessation of MTX, the serum level of soluble interleukin-2 receptor gradually decreased and returned to the normal range after chemotherapy. On the PET-CT after chemotherapy, the stomach continued to show no FDG avidity (Fig. 3c), while the mediastinum and left chest wall remained unchanged, maintaining slightly increased FDG avidity (Fig. 3f) over follow-up of more than 1 year. We subsequently concluded that the FDG-avid spots in the mediastinum and left chest wall were RA-related inflammatory lesions.

## Discussion

Several reports have shown that patients with RA are at increased risk of developing LPD [2], and MTX, the anchor drug of RA, has been recognized as a major cause of LPD. Moreover, recent reports have focused on the clinical importance of MTX-related LPD, as the number of patients with RA treated with MTX are increasing. Approximately 40–50 % of MTX-related LPD occurs in extra-nodal sites, and the gastrointestinal tract, skin, liver, lung, and kidney are reported to be susceptible. However, MTX-related LPD of the stomach is uncommon.

Satoh et al. [6] reported the first case of spontaneous remission of MTX-associated gastric LPD after discontinuation of MTX therapy. However, while some reports have shown spontaneous regression after withdrawal of MTX in MTX-related LPD, regrowth has been observed in some cases. Whether cessation of MTX therapy alone would lead to complete regression is unclear. In the present case, cessation of MTX therapy led to disease regression, and regression was clearly demonstrated with the use of follow-up endoscopy and PET-CT for the duration of medical follow-up of 6 months.

In our case, however, the FDG-avid spots in the mediastinum and left chest wall remained unchanged on follow-up PET-CT 6 months after cessation of MTX. Although ultrasonography-guided fine needle aspiration biopsy specimen of the chest wall nodule showed no abnormal lymphoid cell proliferation, we decided to administer chemotherapy, considering the possibility of residual lesions after cessation of MTX. One year after chemotherapy, and over the following 1-year period, FDG-avid spots in the mediastinum and left chest wall remained unchanged on PET-CT. We eventually concluded that the spots were associated with RA-related inflammatory changes. Inflammatory lesions are well known to show increased uptake of FDG and can cause false-positive PET scan results. On PET-CT, a rheumatoid nodule may appear similar to a malignant soft tissue tumor [7], thus limiting the value of PET-CT for the evaluation of MTX-related LPD in patients with RA. In patients with MTX-related LPD, termination of MTX may be a suitable option initially, whereas its long-term effect is not well established.

Ichikawa et al. [8] recently reported that spontaneous regression was observed in 59 % of patients in whom MTX was withdrawn, and the regression rate was associated with EBV positivity and non-DLBCL histological type . The authors concluded that histology, EBV positivity, and clonality were important predictors of disease progression and regression, and that age (>70 years) and DLBCL histological type were predictive factors of shorter survival.

In patients with MTX-related LPD, the hyperimmune state of RA itself or the MTX-induced immunosuppressive state are thought to provide the basis for the development of LPD, and EBV is thought to play an important role in LPD development. Indeed, approximately 30–50 % of LPD in RA patients treated with MTX is reported to be EBV-positive [4, 5]. In our case, however, EBER demonstrated no positivity in the majority of atypical lymphoid cells. In the present case, with regard to the predictive factors described above, the negative EBER result indicated poor efficacy of the cessation of MTX in disease regression, and age (over 70 years) and histology (DLBCL) indicated shorter survival. However, contrary to our expectations, even despite many reported cases of immunodeficiency-associated LPD, including MTX-related LPD, showing a tendency toward rapid clinical progression [9], we observed disease regression following the cessation of MTX. Moreover, throughout a 1-year period of observation after additional chemotherapy, complete remission was maintained, with no finding of disease recurrence.

As an MTX-induced immunosuppressive state and subsequent EBV reactivation are well described as a basis for the development of MTX-related LPD, cessation of MTX is a suitable therapeutic option in patients with MTX-related “EBER-positive” LPD. In patients with MTX-related “EBER-negative” LPD, however, the effects of cessation of MTX on disease regression is largely unknown, although efficacy has been shown in a fair number of cases. In patients with MTX-related “EBER-negative” LPD, cessation of MTX rather than immediate treatment with chemotherapy may be an option, but only with careful follow-up. When disease regression is not observed after cessation of MTX, prompt administration of chemotherapy must be considered. With regard to MTX-related “EBER-negative” LPD, further study is needed to determine appropriate treatment of this disorder.

In conclusion, we describe here a case of MTX-related gastric LPD, in which disease regression was achieved after cessation of MTX. In RA patients presenting atypical lymphoid cell proliferation, a careful history must be obtained, and “MTX-related LPD” must be considered in patients who were treated with MTX. For those with MTX-related LPD, cessation of MTX may be a therapeutic option, but careful follow-up and chemotherapy in accordance with the clinical course are essential.
